# Protocol of an open-label safety and feasibility pilot study of ketamine-assisted psychotherapy for methamphetamine use disorder (the KAPPA trial)

**DOI:** 10.1136/bmjopen-2024-092504

**Published:** 2025-02-10

**Authors:** Kathryn Fletcher, Nadine Ezard, Krista J Siefried, Harriet MacDonald, Liam Acheson, Gillinder Bedi, Alexandre Guerin, Elizabeth Knock, Michael Millard, Robert May, Jonathan Brett, Jess Doumany, Celia Morgan, Brendan Clifford

**Affiliations:** 1 The National Centre for Clinical Research on Emerging Drugs; The National Drug and Alcohol Research Centre, University of New South Wales, Sydney, New South Wales, Australia; 2 Alcohol and Drug Service, St Vincent's Hospital Sydney, Darlinghurst, New South Wales, Australia; 3 Centre for Youth Mental Health, The University of Melbourne, Melbourne, Victoria, Australia; 4 Substance Use Research Group, Orygen Ltd, Parkville, Victoria, Australia; 5 Clinical Research Unit for Anxiety and Depression, St Vincent's Hospital Sydney, Darlinghurst, New South Wales, Australia; 6 St Vincent's Clinical School; School of Population Health, University of New South Wales, Sydney, New South Wales, Australia; 7 Clinical Pharmacology and Therapeutics, St Vincent's Hospital Sydney, Darlinghurst, New South Wales, Australia; 8 Australian Injecting and Illicit Drug Users League, Canberra, Canberra, Australia; 9 Department of Psychology, University of Exeter, Exeter, Devon, UK; 10 Imperial College London, London, UK

**Keywords:** Substance misuse, Psychosocial Intervention, Drug Therapy, Feasibility Studies, Safety

## Abstract

**Introduction:**

Methamphetamine use disorder is a significant public health concern. No pharmacological treatment options currently exist for methamphetamine use disorder, and psychotherapy is only moderately effective. Preliminary evidence suggests that ketamine-assisted psychotherapy produces sustained improvements in substance use and mental health symptomatology. In addition to direct antidepressant properties, ketamine is hypothesised to increase synaptogenesis and facilitate neuroplasticity, in turn prolonging and enhancing the effects of psychotherapy. Given the withdrawal-associated dysphoria and neurocognitive impairments characterising methamphetamine use disorder, ketamine-assisted psychotherapy may improve the efficacy of psychotherapy alone by addressing these features and facilitating therapeutic engagement. This pilot study aims to investigate the safety and feasibility (time taken to recruit sample, proportion of ineligible participants at pre-screening and screening, number of participants who complete four sessions of psychotherapy, retention rate over full duration of study, acceptability of the intervention) of subanaesthetic ketamine in combination with psychotherapy (cognitive behavioural therapy) for adults with methamphetamine use disorder. Changes in methamphetamine use, cravings and withdrawal, quality of life, and treatment satisfaction will also be explored.

**Methods and analysis:**

This is an open-label, single-arm clinical trial. 20 adults meeting DSM-5-TR criteria for methamphetamine use disorder who are seeking to reduce or cease methamphetamine use will be enrolled in the study through a single-site specialist outpatient stimulant treatment service in inner Sydney (St Vincent’s Hospital, Sydney). A 4-week course with three subcutaneous ketamine doses (0.75 mg/kg to 0.9 mg/kg, titrated according to tolerability) at weekly intervals and four sessions of cognitive behavioural therapy (one at treatment initiation and three within 24–48 hours following each ketamine administration session) will be delivered. Safety and feasibility will be assessed over an 8-week period. Secondary outcomes (changes in methamphetamine use, cravings, withdrawal, quality of life and treatment satisfaction) will be assessed over a 24-week period.

**Ethics and dissemination:**

This study has been approved by the St Vincent’s Hospital Human Research Ethics Committee, reference 2023/ETH00530. Study findings will be disseminated through articles in scientific, peer-reviewed journals, and at national and international conferences.

**Trial registration number:**

ANZCTR: ACTRN12624000895583.

**Protocol version:**

The trial protocol (Version 4.0) was approved on 24 June 2024.

**STRENGTHS AND LIMITATIONS OF THIS STUDY**

- The KAPPA pilot study is the first to examine the safety and feasibility of ketamine-assisted psychotherapy for methamphetamine use disorder.
- Structured clinical intervention of standardised manual-based cognitive behavioural therapy alongside a rigorous low-cost subcutaneous racemic ketamine dosing protocol allows for ready reproducibility.
- Moving beyond abstinence, the study tests the feasibility of measuring validated secondary outcomes that align with harm-reduction and person-centred approaches, including reduction in methamphetamine use and improved quality of life.
- A strength of the study is the use of structured clinical interviews to screen and characterise the sample.
- There is a risk of high attrition rates over the course of the study.

## Introduction

### Background and rationale

 Methamphetamine is the most widely consumed synthetic stimulant drug worldwide,^1^ representing a significant public health concern. Frequent methamphetamine use is associated with a variety of health problems including mental health conditions, insomnia, cardiovascular conditions, cognitive deficits and a risk of drug-induced psychosis.^2 3^ Methamphetamine use disorder (MAUD) often involves increasing tolerance to methamphetamine and continued use to avoid the withdrawal symptoms that follow abrupt cessation.^4^ In 2020–2021 in Australia, methamphetamine accounted for 8.2% of all drug-related hospitalisations and is associated with an increasing rate of drug-related deaths (almost four times higher in 2022 compared with 2000),^5^ highlighting a need for a greater range of effective treatments for this group.

No regulatory-approved pharmacological treatment options currently exist for MAUD. Evidence to date for candidate therapeutic agents from a variety of drug classes is promising but limited.^6^,^11^ The current standard of care therefore relies on psychosocial interventions, primarily cognitive behavioural therapy-based approaches,^12^ which show modest effectiveness.^13^ Relapse to methamphetamine use rates is high, with only one-quarter of individuals remaining abstinent after 1 year following residential rehabilitation.^14^ Further, treatment outcomes differ depending on levels of use, with poorer outcomes observed in people who use methamphetamine frequently compared with those who use less frequently.^15^ Limited efficacy may be due in part to psychosocial interventions not sufficiently addressing cravings and the presence of negative affect during withdrawal, leading to relapse,^16^ or common neurocognitive difficulties observed in MAUD including executive dysfunction, poor working memory, impulsivity and decision-making difficulties.^17^ Supporting psychological interventions with adjuvant pharmacotherapy may overcome some of these challenges, an approach which is beginning to show promise with other substance use disorders.^18^

Combined treatment approaches using ketamine and different forms of psychotherapy (referred to as ketamine-assisted psychotherapy; KAP) have shown sustained improvements in treatment outcomes for a variety of mental health and substance use disorders.^19 20^ Ketamine is a well-characterised dissociative anaesthetic, acting primarily through antagonism of the glutamatergic n-methyl-d-aspartate (NMDA) receptor. Racemic ketamine is used therapeutically as an anaesthetic agent and analgesic (administered parenterally) and in its s-enantiomer form as esketamine, administered intranasally for treatment-resistant depression. Glutamatergic dysregulation has been implicated in depression.^21^,^24^ Subanaesthetic doses of ketamine induce rapid reversal of depression symptoms that can be sustained for up to 7 days after one infusion.^23 25^ The antidepressant properties of ketamine are of particular interest for amphetamine use disorders given findings that chronic amphetamine administration results in depletion of dopamine receptors and dopamine release, leading to a negative affective state during withdrawal,^16^ and increasing the risk of relapse.^26^ While there is significant heterogeneity in how ketamine has been administered in clinical studies to date (eg, intravenous infusion vs subcutaneous injection, dosage, course of treatment), preliminary data suggest that subanaesthetic doses of ketamine may have efficacy for treatment of substance use disorders. Ketamine monotherapy for cocaine, heroin and alcohol use disorder has produced short-lived improvements in self-administration and abstinence compared with active placebo.^20 27 28^ While ketamine provides a unique, rapid onset of antidepressant action for chronic mental health conditions including substance use disorders, its therapeutic effect is temporally limited. Symptom reductions are frequently transient (typically lasting 4–7 days), with repeated administrations required to maintain positive effects.^29^,^31^

In KAP approaches, it is hypothesised that ketamine may prolong and enhance the effect of psychotherapy by increasing synaptogenesis and promoting neural pathways which facilitate neuroplasticity.^19 20^ Ketamine-enhanced neuroplasticity is thought to facilitate emotional learning, evoke emotionally arousing phenomenological experiences, reduce defensiveness and enhance treatment adherence and engagement.^19^ While no studies to date have examined KAP for MAUD specifically, promising findings for other substance use disorders have been reported. A small randomised controlled trial (RCT; n=55) of ketamine-assisted (0.5 mg/kg single intravenous infusion) mindfulness-based relapse prevention therapy for cocaine dependence showed 48.2% abstinence compared with 10.7% in the active control group (midazolam+mindfulness-based relapse prevention therapy), with the odds of end-of-study abstinence in the ketamine group being six times that in the midazolam group (OR=5.7, ᵡ^2^=5.34, df=1, p=0.02). Further, those in the ketamine group were 53% less likely to relapse or discontinue treatment compared with controls over the 5-week study period.^32^ An RCT (n=40) examining ketamine-assisted (0.71 mg/kg single intravenous infusion) motivational enhancement therapy for alcohol use disorder reported a lower proportion of participants in the ketamine group using alcohol (8/17, 47.1%) compared with controls (13/22; 59.1%) across the 21 days following drug administration.^33^ Sustained effects have been reported in alcohol use disorder, whereby those receiving ketamine-assisted (three 0.8 mg/kg intravenous infusions once weekly) mindfulness-based relapse prevention therapy had more days abstinent over 6 months compared with three other groups (ketamine+psychoeducation; placebo+mindfulness-based relapse prevention therapy; placebo+psychoeducation) (mean difference=10.1, 95% CI=1.1, 19.0).^34^ Longer-term effects in other substance use disorders have also been reported. An RCT (n=70) in people with heroin dependence using both a high (2.0 mg/kg) and low (0.2 mg/kg) intramuscular ketamine dose and existential-oriented psychotherapy reported higher rates of abstinence for the high-dose group vs low-dose group, with effects sustained over 24 months.^35^

Supporting the putative impact of ketamine in enhancing effects of psychotherapy more broadly, positive effects of ketamine-assisted cognitive behavioural therapy (CBT) in treatment-resistant depression have also been reported, with CBT sustaining the antidepressant effects of intravenous ketamine (six doses, 0.5 mg/kg, over 3 weeks) over a 17-week period (Quick Inventory of Depressive Symptomatology-Self-Report scores F*=*4.58, p=0.033; d=0.71, 95% CI –0.30 to 1.70).^36^ Furthermore, improvements in cognitive control (eg, executive control, including updating and maintenance) were associated with clinical improvement following ketamine treatment in a subset of patients. This provides some support for the hypothesis that, along with its rapid antidepressant properties, ketamine may also improve cognitive control, while CBT strengthens and maintains these improvements.^36^ A combined approach of ketamine and CBT may therefore have strong potential for MAUD given the neurocognitive impairments and withdrawal-associated dysphoria that appear to hamper psychotherapy effectiveness in this population.

### Objectives

This pilot study aims to determine the safety and feasibility of subcutaneously administered racemic ketamine (three doses, once per week) alongside four sessions of weekly CBT for adults with MAUD, in an outpatient setting. We hypothesise that ketamine-assisted psychotherapy will be safe and feasible in this context.

Secondary objectives are to explore changes from baseline to weeks 5, 8 12 and 24 in methamphetamine use, cravings and withdrawal and quality of life, as well as treatment satisfaction.

Study results will inform future randomised controlled trials.

## Methods and analysis

### Study setting and design

This study is an open-label, single-arm pilot clinical trial. This paper reports on the study protocol in line with Standard Protocol Items Recommendations for Interventional Trials (SPIRIT) guidance (online supplemental file 1).^37^

The study will be conducted in an outpatient stimulant treatment clinic (the ‘Stimulant Treatment Programme’; STP) at St Vincent’s Hospital, Sydney (SVHS), Australia. SVHS, a public teaching hospital, is the study sponsor. Participants will be referred directly from the clinic. Advertising to local health service providers and social media will also be used to enhance recruitment.

### Patient and public involvement

This study is designed and conducted without any involvement from participants or the public. Study results will be disseminated to participants and the public via the National Centre for Clinical Research in Emerging Drugs website.

### Participants and recruitment

#### Eligibility criteria

Eligible participants will be adults who meet DSM-5-TR criteria for MAUD and are seeking treatment to cease or reduce methamphetamine use. Inclusion and exclusion criteria are outlined in table 1.

**Table 1:** Eligibility criteria

Inclusion criteria	Exclusion criteria
All participants must/must be:	All participants must not:
≥18 years of age Able to provide informed consent Willing and able to comply with all study requirements, as determined by the principal investigator Meets DSM-5-TR diagnostic criteria for current stimulant use disorder—amphetamine-type substance—as determined by the principal investigator and confirmed with the MINI Urine drug screen (UDS) point of care (POC) test positive for methamphetamine Willing to register as a client of the St Vincent’s Hospital Sydney (SVHS) Stimulant Treatment Programme (STP)	DSM-5-TR diagnosis of current or past use disorder for ketamine or ketamine analogues as assessed by MINI Prescribed or non-prescribed use of ketamine in the previous 4 weeks Currently enrolled in another treatment trial of MAUD or clinical trial which is likely to affect safety, data quality or may interfere with participation in this study, as determined by the principal investigator Currently pregnant or breastfeeding, or planning on becoming pregnant during the course of the study DSM-5-TR diagnosis of current psychotic disorder as assessed by the principal investigator including review of MINI Current acute suicidality defined as ‘high risk’ using the C-SSRS-6 screener or as determined by the principal investigator DSM-5-TR diagnosis of bipolar disorder as assessed by the principal investigator including review of MINI Current DSM-5-TR diagnosis with other substance use disorders, moderate or severe, except tobacco, caffeine or cannabis as assessed by the principal investigator including review of MINI. Opioid use disorder permitted if stable on opioid agonist treatment; OAT) (ie, no dose changes for 6 weeks if on oral OAT and maximum of one missed dose/week. At least 3 months with no missed doses if on long-acting injectable OAT) History of sensitivity to ketamine or any other components of this product If prescribed antidepressants, the participant must have been on a stable dose for four or more weeks Contraindications to ketamine according to Australian Product Information: Severe cardiovascular disease Heart failure Severe or poorly controlled hypertension Recent myocardial infarction History of stroke Cerebral trauma Intracerebral mass or haemorrhage
Seeking treatment to cease or reduce methamphetamine use If person of childbearing potential, willing to avoid pregnancy for study duration	Any other medical or psychiatric condition which in the opinion of the principal investigator would make participation hazardous. In particular, caution if severe liver, kidney or bladder disease, and also caution if elevated cerebrospinal fluid pressure, increased intraocular pressure, acute intermittent porphyria, seizures, hyperthyroidism, pulmonary or upper respiratory infection, intracranial mass lesions, a presence of head injury, globe injuries, or hydrocephalus Likely or planned surgery, travel, incarceration or other engagement during the study that may interfere with study participation

MAUDmethamphetamine use disorder

#### Participant timeline

**Figure 1:**
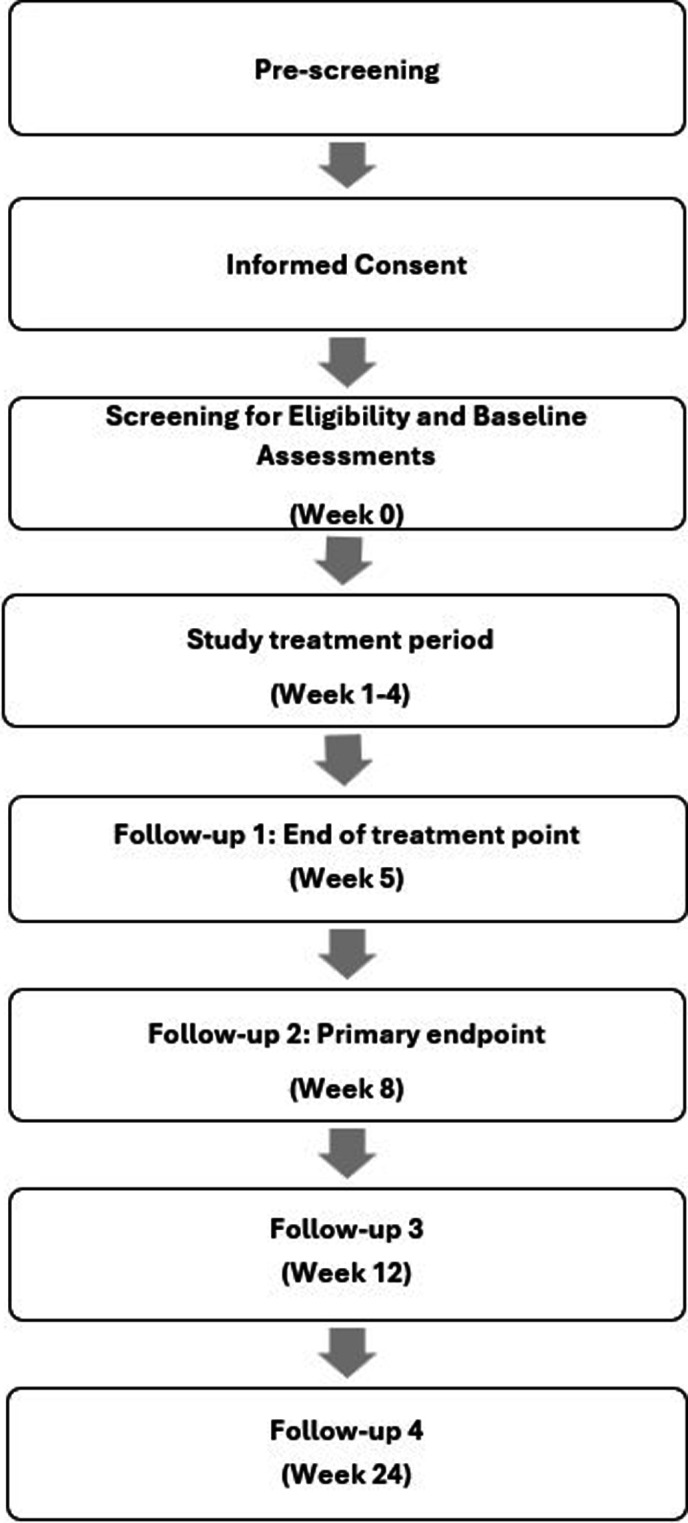
Trial timelines.

Figure 1 gives an overview of the flow of participants through the study. The total study duration for each participant is a maximum of 168 days. This comprises a screening period (window of up to 2 weeks), a 4-week treatment period, end of treatment (week 5), primary endpoint at week 8 and two additional follow-up visits (week 12, week 24).

Potential participants who express interest in the study will be pre-screened and those who meet basic criteria will proceed to informed consent. Informed consent (online supplemental file 2) will be obtained from each participant by the principal investigator or delegated medical officer. Participants will then be formally screened for eligibility. Screening for eligibility will include medical review and a structured clinical interview (the MINI International Neuropsychiatric Interview 7.0.2; MINI)^38^ for substance use disorders, major depressive disorder, bipolar disorder and psychosis. The Columbia Suicide Severity Rating Scale Screener (CSSRS-6)^39^ will be used to assess for history of suicidal ideation or suicide attempts. Eligible participants will be enrolled in the study. Baseline assessments to characterise the sample include demographic information, the Wender Utah Rating Scale (WURS)^40^ to retrospectively evaluate the presence of childhood ADHD symptoms; and the Substance Use Goals and Expectations to measure treatment goals and expectations (SURGE, unpublished). Qualitative interviews (one per participant) will be conducted during weeks 5 to 8. The full schedule of assessments is outlined in online supplemental file 3.

### Intervention

**Figure 2:**
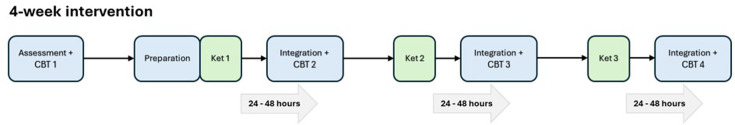
Intervention schedule.

The intervention consists of three subcutaneous racemic ketamine doses at weekly intervals and four sessions of CBT (one at commencement of the intervention and three within 24–28 hours following each ketamine administration session) (see figure 2).

#### Medication

The medication under investigation is the Therapeutic Goods Association (TGA)-approved ketamine 100 mg/mL, available in Australia as a liquid solution formulated for intravenous and intramuscular injection as an anaesthetic agent.^41 42^ A total of three subcutaneous subanaesthetic doses, at least 7 days apart, will be administered by a study nurse to the abdomen once weekly during clinic visits at weeks 2, 3 and 4. The starting dose will be 0.75 mg/kg, increased to a maximum dose of 0.9 mg/kg at subsequent visits as tolerated. The dose can be reduced to 0.6 or 0.5 mg/kg if not tolerated (see table 2).

**Table 2:** Study drug schedule

Ketamine 1	Tolerability	Ketamine 2	Tolerability	Ketamine 3
0.75 mg/kg	Well tolerated	0.9 mg/kg	Well tolerated	0.9 mg/kg
			Moderately tolerated	0.9 mg/kg
			Poorly tolerated	0.75 mg/kg
	Moderately tolerated	0.75 mg/kg	Well tolerated	0.75 mg/kg
			Moderately tolerated	0.75 mg/kg
			Poorly tolerated	0.6 mg/kg
	Poorly tolerated	0.6 mg/kg	Well tolerated	0.6 mg/kg
			Moderately tolerated	0.6 mg/kg
			Poorly tolerated	0.5 mg/kg

Tolerability will be assessed by the study doctor guided by the Ketamine Side Effect Tool (KSET),^43^ with the clinician assigning tolerability prior to discharge based on subjective participant reports and objective observations.^43 44^ Acceptable safety and tolerability profiles have been demonstrated in subcutaneous doses of up to 0.9 mg/kg.^44^ The study nurse will continuously monitor the participant at 120 min post ketamine administration and alert the principal investigator or appropriately qualified delegate in the case of any adverse event of concern, which will be managed according to clinical need (eg, conservatively, continued support for up to a further 120 min).

#### Psychotherapy

The four-session manualised CBT programme developed by Baker *et al*^45^ will be provided by trained therapists to all participants in the trial, with adaptations suitable for the KAP approach. Adaptations are specified in a therapist manual developed specifically for the study (unpublished), detailing ‘set and setting’ considerations within this context, preparation sessions (ie, dosing day discussions including intention-setting, grounding exercises facilitated by the therapist and study nurse) and integration discussions (ie, discussions framing each CBT session in relation to ketamine experiences). For the purposes of this study, therapists are defined as staff with qualifications in a relevant health discipline (such as counselling, nursing, social work, psychology, psychiatry) that have undergone training on the use of the CBT manual and in ketamine-assisted psychotherapy adaptations (2.5-hour training session facilitated by a senior clinical psychologist with experience in CBT and psychedelic-assisted therapy for MAUD). Each psychotherapy session will last approximately 1.5 hours. The initial assessment will take place as part of psychotherapy session 1, reviewing: (1) alcohol and other drug use history, (2) mental health assessment, (3) participant’s readiness to change methamphetamine use, and (4) information about what will happen during the first ketamine dose session. One week later, a preparation session will be undertaken immediately prior to ketamine dose 1. Psychological preparation and pre-injection relaxation exercises have been previously shown to reduce the anxiety and distress that might emerge with ketamine administration.^46^ As in previous studies,^32 47 48^ participants will be guided through grounding exercises (eg, relaxation and breathing) prior to ketamine administration and encouraged to use these should any discomfort or anxiety emerge. As part of preparation, study participants will be briefed on what to expect after drug administration and given the opportunity to ask any questions they may have, and a discharge information sheet will be provided. Within 24–48 hours following ketamine dose 1, psychotherapy session 2 will be undertaken, beginning with an integration focus where experiences of the ketamine administration will be briefly explored and used to guide the subsequent CBT material. This approach will also be used for the remaining sessions. Therapists will co-ordinate the four sessions to occur at least 1 week apart. Session 1 will occur during week 1; sessions 2, 3 and 4 will occur during weeks 2, 3 and 4 respectively within 24–48 hours of participants receiving study medication. Session checklists will be completed post-session by the therapist to assess treatment fidelity and discussed during group supervision with a senior psychologist. As per Baker *et al*,^45^ study participants will be encouraged to complete homework tasks in-between sessions.

### Outcomes

The primary outcomes of this pilot study are safety and feasibility. Safety will be assessed by treatment emergent adverse events (AEs) across the duration of the study, categorised by system organ class (SOC). These will be described by seriousness, severity, causality and expectedness.^49 50^ AEs will be documented at each clinic visit. Subjective descriptions of AEs provided by participants will be transcribed verbatim and reported in accordance with the Medical Dictionary for Regulatory Activities (MedDRA) terminology, developed under the auspices of the International Council for Harmonisation of Technical Requirements for Pharmaceuticals for Human Use (ICH). Severity will be graded from grade 1 (mild) to grade 5 (death) by the site principal investigator.^51^ Causality will be determined by the site principal investigator, and expectedness in accordance with international guidelines and Australian product labels for ketamine hydrochloride 100 mg/mL for injection.^41 42^ Any known reaction listed on the product label will be considered potentially causally related. Adverse events will be elicited through structured assessment with the KSET.^43^ Additional safety measures (see online supplemental file 3 for administration timepoints) include: (i) blood pressure and heart rate during ketamine administration sessions, (ii) dissociative effects as measured by the Clinician-Administered Dissociative States Scale (CADSS-6),^52^ (iii) elevated mood as measured by the Young Mania Rating Scale (YMRS [item 1]),^53^ (iv) suicidality as assessed with the C-SSRS, (v) non-medical use liability as measured by the Drug Effects Questionnaire (DEQ-5),^54 55^ and (vi) changes in other drug use (including ketamine) as measured by Timeline Follow Back (TLFB).^56^

Feasibility will be assessed by: (i) time taken to recruit the sample (with a 12-month study timeframe imposed, based on unpublished studies from our group in similar contexts successfully recruiting 20 participants within this timeframe); (ii) proportion of ineligible participants at pre-screening and screening; (iii) number of participants who receive three doses of ketamine, (iv) number of participants who complete four sessions of psychotherapy, (v) retention rate over the full duration of the study, and (vii) acceptability of the intervention, assessed via qualitative interviews. As this is the first study of its kind in this population and pilot studies are not generalisable to other contexts,^57 58^ these will be assessed and reported descriptively. While we conservatively anticipate 50% completion of 4 sessions of CBT and 75% completion of 6-month follow-up assessments based on studies of CBT in this population,^13 59^ these are not directly comparable given the pharmacotherapy component of the present study design. Therefore, results will be synthesised in consideration of the feasibility to progress to larger studies, taking into account the measures described above and, equally so, the qualitative acceptability.^60^

Secondary outcomes include measures of preliminary efficacy and potential mediators. These measures include: (i) self-reported change in the past 28 days of MA use from baseline to week 5 (post treatment), week 8 (primary endpoint), week 12 and week 24 as measured by the TLFB,^56^ (ii) presence of methamphetamine in urine assessed through POC UDS at week 5, week 8, week 12 and week 24, (iii) changes in methamphetamine craving as measured by the Visual Analogue Scale-Craving (VAS-C)^61^ and withdrawal symptoms assessed on the Amphetamine Withdrawal Questionnaire (AWQ)^62^ from baseline to week 5, week 8, week 12 and week 24, (iv) changes in quality of life as measured by the WHO Quality of Life-Brief Version (WHOQOL-BREF)^63^ from baseline to week 5, week 8, week 12 and week 24, and (v) treatment satisfaction as measured by the Treatment Satisfaction Questionnaire for Medication (TSQM-II)^64^ and the Client Satisfaction Questionnaire (CSQ-8)^65^ at week 5 and week 8.

Potential mediator measures are changes in: (i) depression scores on the Patient Health Questionnaire-9 (PHQ-9)^66^ from baseline to week 5, week 8, week 12 and week 24, (ii) anxiety scores on the Generalised Anxiety Disorder Scale-7 (GAD-7)^67^ from baseline to week 5, week 8, week 12 and week 24, (iii) emotion regulation scores on the Difficulties in Emotion Regulation Questionnaire (DERS)^68^ from baseline to week 5, week 8, week 12 and week 24, (iv) sleep quality scores on the Insomnia Severity Index (ISI)^69^ from baseline to week 5, week 8, week 12 and week 24, (v) HIV and other sexually transmitted infection risk behaviours as measured by the Substance Use Sex Index (SUSI)^70^ from baseline to week 5, week 8, week 12 and week 24, (vi) subjective medication effects on the Hood Mysticism Scale (HMS)^71^ 120 min post ketamine administration, (vii) cognitive control and flexibility as measured by the Emotional N-back task^72^ at baseline and 24 hours post third ketamine administration, and (viii) cognitive control as measured by the Emotional Stroop task^726^ at baseline and 24 hours post third ketamine administration.

### Sample size

The study is not powered to determine efficacy. The study will recruit 20 participants, as is convention in pilot studies.^73^

### Participant retention and withdrawal

The study site will make all reasonable efforts to follow participants for the course of the study. Efforts to minimise loss to follow-up will include respecting participant time commitments, formal tracking procedures including multiple ways to be contacted and strong interpersonal skills of study personnel.

#### Stopping criteria

If a participant experiences a grade 3 or grade 4 adverse event (AE) considered to be causally related to the study medication, no further study medication will be dispensed until the participant has been reviewed by the site principal investigator.^51^ If the AE is resolved to the satisfaction of the principal investigator, the dose can be recommenced, and the participant will be reviewed the subsequent day. If the AE is not resolved or recurs after recommencing the study medication, the site principal investigator will consider ceasing the medication and withdrawing the participant from the treatment component of the study. Unless they revoke their consent, all participants withdrawn from treatment will continue to be followed as intention to treat.

#### Reimbursement

Participants will be reimbursed for participating, in accordance with Australian guidelines for appropriate and equitable payment of participants in research.^74^ Participants who consent to partake in the study and complete all the screening assessments will receive a $40 gift card. Reimbursements of $40 gift cards will also be made for each study visit. If the participant chooses to have their qualitative interview scheduled for the same day as another visit during weeks 5 to 8, they will still receive a separate $40 reimbursement for the interview. The maximum potential amount of reimbursement over the entire duration of the study is $520 of gift cards per person.

### Data management

Study data will be collected and managed using REDCap (Research Electronic Data Capture) tools hosted at St Vincent’s Hospital, Sydney.^75^ REDCap is a secure, web-based software platform designed to support data capture for research studies, providing (i) an intuitive interface for validated data capture, (ii) audit trails for tracking data manipulation, (iii) automated export procedures for seamless data downloads to common statistical packages, and (iv) procedures for data integration and interoperability with external sources. In accordance with the National Standard Operating Procedures for Clinical Trials,^76^ identifiable information will be stored separately from the main study data. The Participant Identification Log will be stored in a password-protected folder on a secure SVHS hosted server. Access to study records within the REDCap database will be limited by using Data Access Groups (DAGs). Only users within a given DAG can access records created by users within that group. Access to components of study records is role-based and can only be granted by the project manager. Data will only be made available to investigators who are directly involved in the collection, analysis or monitoring of study data. Following conclusion of the study, physical and digital records will be stored for a period no less than 15 years as per ICH-GCP guidelines.^49^

### Statistical methods

Descriptive statistics will be used to characterise the study sample. Quantitative analyses will be descriptive and exploratory and include basic measures of central tendency, CIs around means or proportions, Cohen’s d, and paired sample t-tests to assess pre-post intervention changes. P values are considered preliminary and will be reported with caution, supplemented with appropriate metrics (eg, CIs). For categorical measures such as the presence of AEs, rates will be analysed using appropriate non-parametric approaches, such as χ² and relative risk. Quantitative data will be analysed using SPSS Version 29.0.^77^ Qualitative interviews will be collected until all trial participants have been approached. Interviews will be thematically analysed to extract key themes across participant responses, using the approach outlined by Braun & Clarke^78^: familiarising with the data, generating initial codes, searching for themes, reviewing themes, defining and naming themes, and producing the report. Qualitative data will be analysed using NVivo Version 14.^79^

### Monitoring

#### Data Safety Monitoring Board (DSMB)

An independent DSMB will be established prior to study recruitment. DSMB membership will include: an addiction medicine specialist, a psychologist and a pharmacologist (all not otherwise involved with the study). All serious adverse events (SAEs) and suspected unexpected serious adverse reactions (SUSARs) will be reviewed by the DSMB quarterly. Following each meeting, the DSMB will advise one of four options: continue study as per protocol, continue study with protocol amendments, suspend study or discontinue study. The study data will be monitored by a sponsor staff member not otherwise involved in the study, for accuracy, primary endpoint data, and compliance with ICH-GCP^49^ and the Australian National Statement on Ethical Conduct in Human Research.^80^

## Ethics and dissemination

This study has been approved by the St Vincent’s Hospital Human Research Ethics Committee, reference 2023/ETH00530. All participants will provide digital informed consent prior to commencing in the study.

Study findings will be disseminated through articles in scientific, peer-reviewed journals, and at national and international conferences.

## Discussion

This pilot study is the first to examine the safety and feasibility of ketamine-assisted psychotherapy for the treatment of MAUD in adults in an outpatient setting. Secondary outcomes were selected with a harm-reduction and person-centred lens, including reduction in methamphetamine use, cravings, withdrawal symptoms and improved quality of life. As overviewed by Pasareanu *et al*,^81^ substance use disorder treatment has traditionally focused on abstinence but is increasingly incorporating broader positive treatment outcomes. Recovery-oriented outcomes such as quality of life encompass clinical, functional and personal variables, which hold particular relevance for substance use disorder given the chronic nature of the condition. Other study strengths include the use of structured clinical interviews to screen and characterise the sample.

While intranasal and intravenous ketamine represent the most common routes of administration in studies to date,^82^ SC administration was chosen for the current study for several reasons (shorter time of administration, fewer injection-related adverse events, minimal discomfort, cost-effectiveness, and not requiring an anaesthetist for monitoring) and has been used successfully in a large RCT over a 4-week period in adults with treatment-resistant depression,^44^ demonstrating safety and efficacy in doses of up to 0.9 mg/kg. As this is the first published study to our knowledge using SC ketamine in individuals with a substance use disorder, further characterisation of the relative efficacy, tolerability and safety of different routes of administration in this group is needed.

This intervention also offers some promise in terms of scalability, of particular importance given the concerns of the infrastructure required to implement medication-assisted psychotherapy interventions.^83 84^ The 2-hour monitoring period requires both appropriate space and staffing but is shorter than the monitoring required for other medication-assisted psychotherapies, while the subcutaneous route requires less intensive monitoring than intravenous administration. Ketamine for injection is also widely available at relatively low cost. The use of a standardised manual-based CBT also allows for scalability in terms of workforce readiness and reproducibility. Qualitative data from this study will examine the feasibility and acceptability of these components, and future studies will incorporate cost-effectiveness analyses to inform the future delivery of such interventions in real-world settings. Comorbid mental health issues in those who use methamphetamine regularly are common.^10^ The presence of mild to moderate coexisting depression, anxiety or transient psychotic symptoms does not constitute exclusion criteria in the current study to promote real-world application of the findings. Further, successful treatment of MAUD requires consideration of comorbid mental health conditions. Given the co-occurring depressive symptomatology and the withdrawal symptoms of dysphoria and anxiety commonly experienced when ceasing methamphetamine use, ketamine-assisted CBT may have particular efficacy for treating MAUD by addressing these symptoms while enhancing psychotherapeutic effects to prevent relapse.

Extra-medical use of ketamine precipitated by prescribed ketamine may be a cause of concern, especially given increasing prevalence of ketamine use disorder has been observed in some countries/regions.^85^ As overviewed by Le *et al*,^86^ in professionally supervised settings, single or repeated intravenous, intramuscular or oral ketamine administration has not been shown to result in misuse, dependence, diversion and/or gateway activity in patients with treatment-resistant depression. However, extra-medical use liability was not systematically evaluated using validated measures, reports were retrospective in nature, and studies excluded individuals with substance use disorder; thus, results are not directly relevant to this study population. A systematic review of ketamine for the treatment of mental health and substance use disorders found no evidence of transition to extra-medical ketamine use or unexpected psychological complications following treatment with ketamine.^87 88^ The authors concluded that the relatively modest risk of precipitating ketamine use disorder should not present a barrier to treatment. Nonetheless, the safety and efficacy profile of ketamine in those with a substance use disorder requires further investigation. Positively, preliminary evidence supports the potentially beneficial role of ketamine in substance use disorders, with intravenous ketamine in combination with psychosocial treatment reducing alcohol and cocaine craving and consumption.^32^,^34^ The current study represents an important first step in determining whether these findings extend to the MAUD space.

## supplementary material

10.1136/bmjopen-2024-092504 online supplemental file 1

10.1136/bmjopen-2024-092504 online supplemental file 2

10.1136/bmjopen-2024-092504 online supplemental file 3
