# Supplementary material for: Protocol of an open-label safety and feasibility pilot study of ketamine-assisted psychotherapy for methamphetamine use disorder (the KAPPA trial)
Source: BMJ Open. 2025 Feb 10;15(2):e092504. doi: 10.1136/bmjopen-2024-092504 (PMC11815421; doi:10.1136/bmjopen-2024-092504)
Supplement: online supplemental file 2 [file bmjopen-15-2-s002.pdf]

Participant Information Sheet/Consent Form

Interventional Study - *Adult providing own consent*

St Vincent's Hospital, Sydney

**Title**	An Open-Label Safety and Feasibility Pilot Trial of Ketamine-assisted Psychotherapy for Methamphetamine Use Disorder
**Short Title**	Ketamine-assisted Psychotherapy for Methamphetamine Use (KAPPA)
**Protocol Number**	ETH 2024/ETH00530
**Project Sponsor**	St Vincent's Hospital, Sydney
**Principal Investigator**	Professor Nadine Ezard
**Associate Investigator(s)**	Dr Brendan Clifford<br>Dr Krista Siefried<br>A/Professor Gillender Bendi<br>Dr Alexandre Guerin<br>A/Professor Jonathan Brett<br>Dr Michael Millard<br>Dr Robert May<br>Dr Elizabeth Knock<br>Ms Jess Doumany<br>Dr Kathryn Fletcher<br>Mr Liam Acheson
**Location**	St Vincent's Hospital, Sydney, Alcohol and Drug Service

Part 1 What does my participation involve?

1 Introduction

You are invited to take part in this research project. This is because you have indicated that you currently use methamphetamine and are interested in managing, reducing, or stopping your methamphetamine use. The research project is testing a new treatment for methamphetamine use disorder. The new treatment is called ketamine assisted psychotherapy.

This Participant Information Sheet/Consent Form tells you about the research project. It explains the tests and treatments involved. Knowing what is involved will help you decide if you want to take part in the research.

Please read this information carefully. Ask questions about anything that you don't understand or want to know more about. Before deciding whether to take part, you might want to talk about it with a relative, friend or your local doctor.

Participation in this research is voluntary. If you don't wish to take part, you don't have to. You will receive the best possible care whether you take part.

If you decide you want to take part in the research project, you will be asked to digitally sign the consent section on a laptop while you are in the clinic. By signing it you are telling us that you:

- Understand what you have read;
- Consent to take part in the research project;
- Consent to have the tests and treatments that are described; and
- Consent to the use of your personal and health information as described.

You will be emailed a copy of this signed Participant Information Sheet/Consent Form to keep.

2 What is the purpose of this research?

There are currently no approved medications in Australia to help treat methamphetamine dependence. This study aims to determine if it is safe and feasible for people who are using methamphetamine to be given a medication, called ketamine hydrochloride, along with psychological therapy, to help manage, reduce, or stop their methamphetamine use.

Medications, drugs and devices have to be approved for use by the Australian Federal Government. Ketamine hydrochloride is approved in Australia as an anaesthetic agent; however, it is currently not approved to treat methamphetamine use disorder. Therefore, this is an experimental treatment for methamphetamine use disorder. This means that this medication must be tested to see if it is a safe and effective treatment for people with methamphetamine dependence. This medication will be provided alongside a psychological therapy called Cognitive Behaviour Therapy (CBT), which is known to be effective for people with methamphetamine dependence.

This research has been initiated by the study doctor, Professor Nadine Ezard. This research is funded by the National Centre for Clinical Research on Emerging Drugs, University of New South Wales.

3 What does participation in this research involve?

If, after reading this information and discussing this research with your doctor, friends or family, you decide to take part, you will be asked to digitally sign the Consent Form confirming that you agree to participate. This study is designed to provide you with ketamine hydrochloride and psychological therapy, as well as your usual standard of care provided by St Vincent's Hospital, Sydney. This means that except for receiving the study medication and psychological therapy, the medical care that you will receive will be the same whether you decide to take part in this research or not.

<u>Screening and baseline assessments</u>

Before you take part in the study, the researchers need to make sure it is safe and appropriate for you to do so. This will involve some screening procedures, including questionnaires, medical assessments, and urine tests. These procedures are as follows:

- Review of your medical history including previous instances of substance use treatment, psychological therapy, and any medications you are taking currently.
- Routine medical screening, including blood pressure, pulse, temperature, and weight. All of these measures are non-invasive.
- Routine urine tests, including:
  - A dip-stick urine drug test for methamphetamine. This will require approximately 2-3 tablespoons (50mL) of urine.
  - A pregnancy test will also be conducted if you are of childbearing potential.

- A clinical interview to assess your mental health history, including:
  - Substance use
  - Psychosis
  - Depression
  - Bipolar disorder

- A series of baseline questionnaires to assess your:
  - Use of substances (including methamphetamine, ketamine, opioids, alcohol, cigarettes, etc.) over the past 4 weeks
  - Craving for methamphetamine
  - Withdrawal from methamphetamine
  - Thoughts of suicide and acts of suicidal behaviour

- Mood symptoms
- Quality of life
- Sleep
- Coping with feelings
- Sex and drug use risk behaviours
- Care received outside St Vincent's Hospital

These screening and baseline procedures (including study questionnaires, which will take approximately 1.5 hours to complete) are expected to take between two and three hours. These procedures can be completed over more than one clinic visit if necessary, but all procedures must be completed within 14 days of consenting to participate in the study.

<u>Intervention</u>

Medication

This study involves a commercially available medication which contains ketamine hydrochloride. The medication is in liquid form and will be administered subcutaneously (injection under the skin, in the anterior abdominal wall). You will be required to receive three doses of this medication in total, once per week, during clinic visits. The dose will be adjusted at each visit depending on your level of tolerability. You will be asked to complete some study questionnaires, which will take approximately 30 mins to complete. These visits are expected to take between two and three hours in total.

Psychological Therapy

You will be required to attend four sessions of psychological therapy in total, once per week, during clinic visits. The psychological therapy provided in this study is Cognitive Behavioural Therapy (CBT), by a trained health professional. Sessions will focus on:

- Motivation and preparing to change methamphetamine use
- Coping with cravings and lapses
- Controlling thoughts about methamphetamine use
- Preparing for future high-risk situations

Each session is expected to take 1.5 hours.

<u>Weekly clinic visits</u>

After enrolling in the study and completing all the screening and baseline measures, you will be required to attend St Vincent's Hospital, Sydney, Alcohol and Drug Service i) twice weekly for a period of 4 weeks, ii) four visits at week 5, 8, 12, and 24. The purpose of these visits is to provide you with the study medication and psychotherapy (first 4 weeks), monitor you for any medication related side effects, and to test your urine for methamphetamine.

Each week, you will be asked about care received outside St Vincent's Hospital, other medications you are taking, and physical health symptoms. Your blood pressure, pulse, temperature, and weight will be recorded at each weekly visit. You will provide a urine sample for a point of care methamphetamine screen, and for pregnancy if you are of childbearing potential. In addition, every four weeks you will be asked about your drug consumption (methamphetamine, opioids, alcohol, and cigarettes/nicotine, etc). At the study visits on week 5, 8, 12, and 24 you will also be asked to complete many of the same questionnaires that you completed at the start of the study, which will take approximately 1.5 hours to complete.

Interview

We would also like to interview you about your experiences related to taking part in the study. Interviews will be semi-structured, meaning that the interviewer will have questions to ask you, but some questions may be changed or skipped depending on what you wish to talk about. Interviews will be conducted one-on-one with a trained research staff member. The interview will take approximately one hour. The interview will focus on your goals around managing your methamphetamine use, your expectations around taking the study medication and psychotherapy, your experiences of taking the study medication and receiving psychotherapy, any concerns relating to the study medication or psychotherapy, and your experience of taking part in the study in general.

Interviews will be conducted in a private treatment or interview room at St Vincent's Hospital, Sydney. For those unable to attend in person, phone or video options will be provided. Interviews will be conducted between Weeks 5 and 8 of the study. Interviews will be audio recorded and then transcribed (written down) in full. Participation in the interview requires that you consent for your interview to be audio-recorded. If you do not wish to participate in the interview, you are still able to participate in the study trial.

Reimbursement

Participation in this study requires thirteen visits to St Vincent's Hospital, Sydney, Alcohol and Drug Service clinic and one interview with a researcher. Given the time burden imposed by participation in this study, you will be reimbursed for your time and associated expenses at each visit. Reimbursements are a fixed amount and will be made after each weekly visit that you attend. The maximum potential amount of reimbursement over the entire duration of the study is $520 of gift cards per person.

Week	Screening	1	2 (Two visits)	3 (Two visits)	4 (Two visits)	5	8	12	24	Interview
Amount	$40	$40	$40 x 2	$40 x 2	$40 x 2	$40	$40	$40	$40	$40*

4 What do I have to do?

There are no lifestyle restrictions or dietary restrictions associated with this project. You may continue to do everything that you normally would. During the screening process, the study doctor will ask you about the medications that you are currently taking. It is important that you tell the study doctor what medications you are taking to ensure that it is safe for you to participate in the study. We also ask that you inform the study doctor if there are any changes to your medications or substance use at any point during the study.

If you take part in this study, you are asked to commit to attending clinic visits to receive ketamine administration and attend psychotherapy sessions. You should not use methamphetamine or any other substances for at least 12 hours prior to having any of the ketamine administration.

It is desirable that your local doctor (i.e. your GP) be advised of your decision to participate in this research project. If you have a local doctor, we strongly recommend that you inform them of your participation in this research project.

5 Other relevant information about the research project

You will be participating in a single group pilot study. In this study, all participants receive the same treatment. There will be up to 20 participants taking part in this study.

This study has been designed to make sure the researchers interpret the results in a fair and appropriate way and avoids study doctors or participants jumping to conclusions.

There are no additional costs associated with participating in this research project. All medication, tests and medical care required as part of the research project will be provided to you free of charge.

This research involves collaboration with researchers from St Vincent’s Hospital Sydney and the University of New South Wales.

6 Do I have to take part in this research project?

Participation in any research project is voluntary. If you do not wish to take part, you do not have to. If you decide to take part and later change your mind, you are free to withdraw from the project at any stage.

If you do decide to take part, you will be asked to sign this Participant Information and Consent Form electronically and you will be given a copy to keep.

Your decision whether to take part or not to take part, or to take part and then withdraw, will not affect your routine treatment, your relationship with those treating you or your relationship with St Vincent’s Hospital, Sydney.

7 What are the alternatives to participation?

You do not have to take part in this research project to receive treatment at St Vincent’s Hospital. Other options are available; these include counselling and psychosocial support. Your study doctor will discuss these options with you before you decide whether to take part in this research project. You can also discuss these options with your local doctor. If you would like more support with your substance use, please contact:

- The National Alcohol and Other Drug Hotline: 1800 250 015
- St Vincent's Alcohol and Drug Service Centralised Intake line: (02) 8382 1080 (if residing in Sydney)
- St Vincent's Stimulant Treatment Line: (02) 8382 1111
- Lifeline: 13 11 14
- Alcohol and Drug Counselling Online: https://www.counsellingonline.org.au/
- In the case of emergency: 000

This research study differs from standard care for methamphetamine use, as there are currently no medications approved for the treatment of methamphetamine use in Australia.

8 What are the possible benefits of taking part?

We cannot guarantee or promise that you will receive any benefits from this research; however, possible benefits may include reducing or stopping your methamphetamine use. Participation in this research may help to develop effective, evidence-based treatment options for people who use methamphetamine.

9 What are the possible risks and disadvantages of taking part?

Medical treatments often cause side effects. You might have none, some, or all of the effects listed below. These effects may be mild, moderate or severe. If you have any of these side effects, or are worried about them, talk with your study doctor. Your study doctor will also be looking out for side effects at your weekly clinic visits.

Although the side effects associated with ketamine hydrocholoride are well understood, there may be side effects that the researchers do not expect or do not know about, which may be serious. Tell your study doctor immediately about any new or unusual symptoms that you get at any point during the study.

Many side effects go away shortly after treatment ends. However, sometimes side effects can be serious, long lasting, or permanent. If a severe side effect or reaction occurs, your study doctor may need to stop your treatment. Your study doctor will discuss the best way of managing any side effects with you.

The most common side effects associated with ketamine hydrochloride are nausea, vomiting, and increased salivation. Possible side effects of subcutaneous injection include pain at the injection site (which may persist for 38 hours), bruising, or accidental injection into a blood vessel. The injection site will be observed at 120 minutes, prior to discharge and at scheduled reviews, and any complications will be managed accordingly. Other adverse effects of ketamine hydrochloride include:

- Anorexia
- Anaphylaxis
- Rash
- Confusion, excitation, irrational behaviour

- Hallucinations, vivid imagery, dream-like states, nightmares
- Agitation, anxiety or insomnia
- Movements resembling seizures
- Hypertonia
- Breathing difficulties
- Respiratory depression following rapid IV dose
- Laryngospasm and airway obstruction
- Elevated blood pressure, fast or slow heartbeat, heart palpitations, feeling faint
- Hypotension, arrhythmia
- Double vision or abnormal eye movements
- Elevated intraocular pressure measurement
- Changes in urine colour, pain or burning sensations when urinating, frequently passing small amounts of urine or a persistent urge to urinate
- Acute kidney injury, hydronephrosis, ureteral disorder, haemorrhagic cystitis, cystitis reported during long-term use (>1 month)
- Jaundice
- Drug induced liver injury in extended period use (>3d)
- Abnormal liver function test

The effects of ketamine hydrochloride on the unborn child and on the newborn baby are not known. Because of this, it is important that research project participants are not pregnant or breast-feeding and do not become pregnant during the course of the research project. You must not participate in the research if you are pregnant or trying to become pregnant, or breast-feeding. If child-bearing is a possibility for you, you will be required to undergo a pregnancy test prior to commencing the research project. If you can make someone pregnant, you should not make someone pregnant or donate sperm for at least one month after the last dose of study medication.

All participants must avoid pregnancy during the course of the research and for a period of one month after completion of the research project. You should discuss effective methods of avoiding pregnancy with your study doctor.

If you do become pregnant whilst participating in the research project, you should advise your study doctor immediately. Your study doctor will withdraw you from the research project and advise on further medical attention should this be necessary. You must not continue in the research if you become pregnant.

You should advise your study doctor if you have made someone pregnant while participating in the research project. Your study doctor will advise on medical attention for your partner should this be necessary.

If you become upset or distressed as a result of your participation in the research, the study doctor will be able to arrange for counselling or other appropriate support. Any counselling or support will be provided by qualified staff who are not members of the research project team. This counselling will be provided free of charge.

This research project involves the collection of information about your use of drugs. Participation in the research project includes urine analysis to determine the presence of methamphetamine. That information will be stored in a re-identifiable (or coded) format. In the rare event that SVHS is required to disclose that information, it may be used against you in legal proceedings or

otherwise. These legal obligations apply whether you are involved in a research study or not - any time a person engages in healthcare for substance use disorder, there is an inherent risk in exposure of activity as per legal frameworks. That is, participation in this study will not change the legal obligations that SVHS has in relation to information collected from you.

10 What will happen to my test samples?

This study includes the collection of urine. These collections are a mandatory component of the research. The samples that are collected from you are not retained after testing at the point of care and disposed of in the clinic on the same day.

11 What if new information arises during this research project?

Sometimes during the course of a research project, new information becomes available about the treatment that is being studied. If this happens, your study doctor will tell you about it and discuss with you whether you want to continue in the research project. If you decide to withdraw, your study doctor will make arrangements for your regular health care to continue. If you decide to continue in the research project you will be asked to sign an updated consent form.

Also, on receiving new information, your study doctor might consider it to be in your best interests to withdraw you from the research project. If this happens, they will explain the reasons and arrange for your regular health care to continue.

12 Can I have other treatments during this research project?

Whilst you are participating in this research project, you may not be able to take some or all of the medications or treatments you have been taking for your condition or for other reasons. It is important to tell your study doctor and the study staff about any treatments or medications you may be taking, including over-the-counter medications, vitamins or herbal remedies, acupuncture, or other alternative treatments. You should also tell your study doctor about any changes to these during your participation in the research project. Your study doctor should also explain to you which treatments or medications need to be stopped for the time you are involved in the research project.

13 What if I withdraw from this research project?

If you decide to withdraw from the project, please notify a member of the research team before you withdraw. This notice will allow that person or the research supervisor to discuss any health risks or special requirements linked to withdrawing.

If you do withdraw your consent during the research project, the study doctor and relevant study staff will not collect additional personal information from you, although personal information

already collected will be retained to ensure that the results of the research project can be measured properly and to comply with law. You should be aware that data collected by St Vincent's Hospital up to the time you withdraw will form part of the research project results. If you do not want them to do this, you must tell them before you join the research project.

14 Could this research project be stopped unexpectedly?

This research project may be stopped unexpectedly for a variety of reasons. These may include reasons such as:

- Unacceptable side effects
- The medications being shown not to be effective
- The medications being shown to work and not need further testing
- Decisions made by local regulatory/health authorities

15 What happens when the research project ends?

Should you require treatment for methamphetamine dependence in the future, you will be offered the clinic's usual care of counselling and psychosocial support and follow-up. The study drug ketamine hydrochloride is currently not licensed for use outside of clinical trials for the treatment of methamphetamine use in Australia. When the study is finished, you should speak to your Doctor about the treatment options available to you.

If you would like to find out about the results of the research, please advise your study coordinator who will be able to email you a one-page summary of the findings after the analysis is completed.

Part 2 How is the research project being conducted?

16 What will happen to information about me?

By signing the consent form you consent to the study doctor and relevant research staff collecting and using personal information about you for the research project. Any information obtained in connection with this research project that can identify you will remain confidential. While confidentiality cannot be fully assured despite stringent security measures, the researchers will ensure that all processes are in place to avoid a privacy and confidentiality breach. All information that is collected about you for the purposes of this study will be recorded with a code number instead of your name. Your information will only be used for the purpose of this research project and it will only be disclosed with your permission, except as required by law.

Information obtained during the research project are subject to inspection (for the purpose of verifying the procedures and the data) by the relevant authorities and authorised

representatives of the Sponsor, St Vincent's Hospital, Sydney, the institution relevant to this Participant Information Sheet, or as required by law. By signing the Consent Form, you authorise release of, or access to, this confidential information to the relevant study personnel and regulatory authorities as noted above.

It is anticipated that the results of this research project will be published and/or presented in a variety of forums. In any publication and/or presentation, information will be provided in such a way that you cannot be identified.

In accordance with relevant Australian and or New South Wales privacy and other relevant laws, you have the right to request access to your information collected and stored by the research team. You also have the right to request that any information with which you disagree be corrected. Please contact the study team member named at the end of this document if you would like to access your information.

Any information obtained for the purpose of this research project that can identify you will be treated as confidential and securely stored. It will be disclosed only with your permission, or as required by law.

After the study has been completed, all study-related documents will be stored securely for 15 years in line with national research guidelines, and then securely destroyed.

17 Complaints and compensation

If you suffer any injuries or complications as a result of this study, you should contact the study team as soon as possible and you will be assisted in arranging appropriate medical treatment. In the event of loss or injury, the parties involved in this research project have agreed that you may be entitled to seek compensation for any injuries or complications resulting from the study if your injury or complication is sufficiently serious and is caused by unsafe Investigational Product or by the negligence of one of the parties involved in the study (for example, the researcher, the hospital, or the treating doctor). You may wish to seek legal advice to explore your options. You do not give up any legal rights to compensation by participating in this study.

If you receive compensation that includes an amount for medical expenses, you will be required to pay for any medical treatment required for your injury or complication from those compensation monies. If you are not eligible for compensation for your injury or complication under the law, but are eligible for Medicare, then you can seek medical treatment required for your injury or complication free of charge as a public patient in any Australian public hospital. If you are not eligible for Medicare you may be able to claim compensation back via your private health insurance.

18 Who is organising and funding the research?

This research project is being conducted by St. Vincent's Hospital, Sydney and is being funded by the National Centre for Clinical Research on Emerging Drugs (NCCRED), the University of

New South Wales. NCCRED is funded by the Australian Government Department of Health and Aged Care.

St. Vincent’s Hospital, Sydney, may benefit financially from this research project if, for example, the project assists St. Vincent’s Hospital, Sydney to obtain approval for a new drug.

You will not benefit financially from your involvement in this research project even if, for example, your samples (or knowledge acquired from analysis of your samples) prove to be of commercial value to St. Vincent’s Hospital, Sydney.

In addition, if knowledge acquired through this research leads to discoveries that are of commercial value to St. Vincent’s Hospital, Sydney, the study doctors or their institutions, there will be no financial benefit to you or your family from these discoveries.

St. Vincent’s Hospital, Sydney will receive a payment from the University of New South Wales, National Centre for Clinical Research on Emerging Drugs for undertaking this research project.

No member of the research team will receive a personal financial benefit from your involvement in this research project (other than their ordinary wages).

19 Who has reviewed the research project?

All research in Australia involving humans is reviewed by an independent group of people called a Human Research Ethics Committee (HREC). The ethical aspects of this research project have been approved by the HREC of St Vincent’s Hospital, Sydney.

This project will be carried out according to the *National Statement on Ethical Conduct in Human Research (2023)*. This statement has been developed to protect the interests of people who agree to participate in human research studies.

20 Further information and who to contact

The person you may need to contact will depend on the nature of your query.

If you have any medical problems which may be related to your involvement in the project (for example, any side effects), you can contact the principal study doctor (clinical contact person) as follows:

Clinical contact person

Name	Nadine Ezard
Position	Principal Investigator
Telephone	02 8382 1111
Email	Nadine.Ezard@svha.org.au

If you have any general enquiries about the study or want any further information concerning this study, you can contact the general enquiries contact person as follows:

General enquiries contact person

Name	Lucy Flood
Position	Clinical Trial Coordinator
Telephone	<<telephone number>>
Email	Lucy.flood@svha.org.au

For matters relating to research at the site at which you are participating, the details of the local site complaints person are:

Complaints contact person

Name	Research Office Manager
Position	Research Office Manager
Telephone	02 8382 4960
Email	svhs.research@svha.org.au

If you have any complaints about any aspect of the project, the way it is being conducted or any questions about being a research participant in general, then you may contact:

Reviewing HREC approving this research and HREC Research Officer details

Reviewing HREC name	St Vincent's Hospital, Sydney HREC
Position	Research Officer
Telephone	02 8382 4960
Email	svhs.research@svha.org.au

Local HREC Office contact (Single Site -Research Governance Officer)

Name	Research Governance Officer
Position	Research Governance Officer
Telephone	02 8382 4960
Email	svhs.research@svha.org.au

Consent Form - *Adult providing own consent*

**Title**	An Open-Label Safety and Feasibility Pilot Trial of Ketamine-assisted Psychotherapy for Methamphetamine Use Disorder
**Short Title**	Ketamine-assisted Psychotherapy for Methamphetamine Use (KAPPA)
**Protocol Number**	ETH 2024/ETH00530
**Project Sponsor**	St Vincent's Hospital, Sydney
**Coordinating Principal Investigator/ Principal Investigator**	Professor Nadine Ezard
**Associate Investigator(s)**	Dr Brendan Clifford<br>Dr Krista Siefried<br>A/Professor Gillender Bendi<br>Dr Alexandre Guerin<br>A/Professor Jonathan Brett<br>Dr Mike Millard<br>Dr Robert May<br>Dr Elizabeth Knock<br>Ms Jess Doumany<br>Dr Kathryn Fletcher<br>Mr Liam Acheson
**Location**	St Vincent's Hospital, Sydney, Alcohol and Drug Service

<u>Declaration by Participant</u>

I have read the Participant Information Sheet or someone has read it to me in a language that I understand.

I understand the purposes, procedures and risks of the research described in the project.

I give permission for my doctors, other health professionals, hospitals or laboratories outside this hospital to release information to St Vincent's Hospital, Sydney concerning my disease and treatment for the purposes of this project. I understand that such information will remain confidential.

I consent to participate in the optional qualitative interview and for the interview to be audio-recorded:

☐ Yes ☐ No

I have had an opportunity to ask questions and I am satisfied with the answers I have received.

I freely agree to participate in this research project as described and understand that I am free to withdraw at any time during the study without affecting my future health care.

I understand that I will be given a signed copy of this document to keep.

Name of Participant (please print) ______________________________	
Signature ______________________	Date ______________________

Declaration by Study Doctor/Senior Researcher†

I have given a verbal explanation of the research project, its procedures and risks and I believe that the participant has understood that explanation.

Name of Study Doctor/<br>Senior Researcher† (please print) ______________________________	
Signature ______________________	Date ______________________

† A senior member of the research team must provide the explanation of, and information concerning, the research project.

Note: All parties signing the consent section must date their own signature.

ST VINCENT'S HOSPITAL
SYDNEY
A FACILITY OF ST VINCENT'S HEALTH AUSTRALIA

Form for Withdrawal of Participation - *Adult providing own consent*

**Title**	An Open-Label Safety and Feasibility Pilot Trial of Ketamine-assisted Psychotherapy for Methamphetamine Use Disorder
**Short Title**	Ketamine-assisted Psychotherapy for Methamphetamine Use (KAPPA)
**Protocol Number**	ETH 2024/ETH00530
**Project Sponsor**	St Vincent's Hospital, Sydney
**Principal Investigator**	Professor Nadine Ezard
**Associate Investigator(s)**	Dr Brendan Clifford<br>Dr Krista Siefried<br>A/Professor Gillender Bendi<br>Dr Alexandre Guerin<br>A/Professor Jonathan Brett<br>Dr Mike Millard<br>Dr Robert May<br>Dr Elizabeth Knock<br>Ms Jess Doumany<br>Dr Kathryn Fletcher<br>Mr Liam Acheson
**Location**	St Vincent's Hospital, Sydney, Alcohol and Drug Service

Declaration by Participant

I wish to withdraw from participation in the above research project and understand that such withdrawal will not affect my routine treatment, my relationship with those treating me or my relationship with St Vincent's Hospital, Sydney.

Name of Participant (please print)	____________________
Signature ____________________	Date ____________________

Declaration by Study Doctor/Senior Researcher†

I have given a verbal explanation of the implications of withdrawal from the research project and I believe that the participant has understood that explanation.

Name of Study Doctor/<br>Senior Researcher† (please print)	____________________
Signature ____________________	Date ____________________

† A senior member of the research team must provide the explanation of and information concerning withdrawal from the research project.
