# Supplementary material for: Protocol of an open-label safety and feasibility pilot study of ketamine-assisted psychotherapy for methamphetamine use disorder (the KAPPA trial)
Source: BMJ Open. 2025 Feb 10;15(2):e092504. doi: 10.1136/bmjopen-2024-092504 (PMC11815421; doi:10.1136/bmjopen-2024-092504)
Supplement: online supplemental file 3 [file bmjopen-15-2-s003.pdf]

Supplemental File 3 - Schedule of Assessments

**Week**	**0**	**1**	**2**		**3**		**4**		**5**	**8***	**12**	**24**
**Visit Number**	1	2	3	4	5	6	7	8	9	10	11	12
**Day**	0	1	5/6	7	12/13	14	19/20	21	28	56	84	168
Intervention		CBT 1	KET 1	CBT 2	KET 2	CBT 3	KET 3	CBT 4				
Informed Consent	x											
MINI[a]	x											
C-SSRS-6	x											
Medical assessment	x											
Concomitant Medications	x		x		x		x		x	x	x	x
Other Psychological care	x		x		x		x		x	x	x	x
Height, weight	x		x		x		x					
Vital signs[b]	x		$x^{0, 15, 60, 120}$		$x^{0, 15, 60, 120}$		$x^{0, 15, 60, 120}$		x	x	x	x
POC – Urine Drug Screen[c]	x		x^{PRE}		x^{PRE}		x^{PRE}		x	x	x	x
Urinary Hcg (if applicable)	x		x^{PRE}		x^{PRE}		x^{PRE}		x	x	x	x
Eligibility	x											
Demographics	x											
SURGE	x											
WURS	x											
TLFB - MA	x		x		x		x		x	x	x	x
VAS-C for MA	x		x		x		x		x	x	x	x
AWQ	x								x	x	x	x
WHOQOL-BREF	x								x	x	x	x
TSQM-II									x	x		
CSQ-8									x	x		
Medical Review			x		x		x			x		
Adverse Events		x	x	x	x	x	x	x	x	x	x	x
C-SSRS-SLV			x		x		x		x	x	x	x
TLFB – ketamine	x		x		x		x		x	x	x	x
TLFB – other substances	x								x	x	x	x
VAS-C for Ketamine	x		x		x		x		x	x	x	x
KSET – acute treatment			$x^{60, 120}$		$x^{60, 120}$		$x^{60, 120}$					
YMRS			$X^{PRE,POST}$		x^{POST}		x^{POST}					
CADSS-6			X^{POST}		x^{POST}		x^{POST}					
DEQ-5			x^{POST}		x^{POST}		x^{POST}					
HMS			x^{POST}		x^{POST}		x^{POST}					
PHQ-9	x								x	x	x	x
GAD-7	x								x	x	x	x
DERS	x								x	x	x	x
ISI	x								x	x	x	x
SUSI	x								x	x	x	x
Emotional N-back task	x							x^{PRE}				
Emotional Stroop	x							x^{PRE}				
Qualitative Interview[d]									x			

***Primary Endpoint **AWQ** Amphetamine Withdrawal Questionnaire **CADSS-6** Clinician Administered Dissociation States Scales **C-SSRS** Columbia-Suicide Severity Rating Scale **C-SSRS** Columbia-Suicide Severity Rating Scale – Since Last Visit **CSQ-8** Client Satisfaction Questionnaire **DERS** Difficulties in Emotion Regulation Scale **DEQ-5** Drug Effects Questionnaire **GAD-7** Generalised Anxiety Disorder Scale-7 **HMS** Hood Mysticism Scale **ISI** Insomnia Severity Index **KSET** Ketamine Side Effect Tool **MA** Methamphetamine **MINI** Mini-International Neuropsychiatric Interview **PHQ-9** Patient Health Questionnaire **POC** Point of Care **SURGE** Substance Use Goals and Expectations **SUSI** Substance Use & Sex Index **TLFB** Timeline Follow-Back **TSQM-II** Treatment Satisfaction Questionnaire for Medication

VAS-C Visual Analogue Scale – Craving **WURS** Wender-Utah Rating Scale **WHOQOL-BREF** World Health Organisation Quality of Life Brief Version **YMRS** Young Mania Rating Scale

(a) MINI modules A (major depressive disorder), C (bipolar disorder), I and J (alcohol and substance use disorders), K (current psychotic disorder), 0 (rule out medical, organic or drug causes), (b) Blood pressure, heart rate, oxygen saturations, respiratory rate (c) POC also considered a measure of efficacy (d) Qualitative interviews (optional) will be conducted during Week 5 to Week 8.
