# Supplementary material for: Protocol of an open-label safety and feasibility pilot study of ketamine-assisted psychotherapy for methamphetamine use disorder (the KAPPA trial)
Source: BMJ Open. 2025 Feb 10;15(2):e092504. doi: 10.1136/bmjopen-2024-092504 (PMC11815421; doi:10.1136/bmjopen-2024-092504)
Supplement: Supplementary file 4 [file bmjopen-2024-092504.reviewer_comments.pdf]

PEER REVIEW HISTORY

BMJ Open publishes all reviews undertaken for accepted manuscripts. Reviewers are asked to complete a checklist review form and are provided with free text boxes to elaborate on their assessment. These free text comments are reproduced below.

ARTICLE DETAILS

Title (Provisional)

Protocol of an open-label safety and feasibility pilot study of ketamine-assisted psychotherapy for methamphetamine use disorder (the KAPPA trial)

Authors

Fletcher, Kathryn; Ezard, Nadine; Siefried, Krista J; MacDonald, Harriet; Acheson, Liam; Bedi, Gillinder; Guerin, Alexandre; Knock, Elizabeth; Millard, Michael; May, Robert; Doumany, Jess; Clifford, Brendan; Brett, Jonathan.; Morgan, Celia

---

VERSION 1 - REVIEW

**Reviewer**	**1**
**Name**	**Tsui, Judith**
**Affiliation**	**University of Washington, Medicine**
**Date**	**28-Sep-2024**
**COI**	**None**

This is a protocol paper describing an open label, single arm clinical trial of subcutaneous (SC) ketamine combined with cognitive behavioral therapy (CBT) weekly for one month for treatment of methamphetamine use disorder. Main outcomes are safety and feasibility measured over an 8-week period; secondary outcomes (including methamphetamine use, craving, withdrawal, quality of life and treatment satisfaction). The study addresses a clinical area of high need: methamphetamine use is a problem world-wide and effective treatments are needed. The focus on ketamine is timely. There are currently no regulatory-approved pharmacologic treatment options for methamphetamine use disorder (MUD) and there is increasing interest in ketamine as treatment for a wide variety of mental health and substance use disorders. The authors provide rationale to study ketamine for MUD given that it leads to dopamine depletion and depression and that there are a handful of small studies a ketamine monotherapy for depression, cocaine, heroin and alcohol use that suggest benefit. They provide rationale for combining with CBT arguing for ketamine-induced neuroplasticity that may enhance effects of CBT. The study design is well-conceived and clearly described, although there are some questions about the selection of the outcomes that need clarification. The protocol is detailed and there are helpful figures and

tables to organize details of the study and orient the reader. One major limitation pertains to the delivery of the intervention and assurance of safety/comfort of research participants. Additionally, there are minor criticisms of writing and framing of the study intervention and its potential for scalable. Overall, criticisms should be addressable. I believe that this study protocol, and eventually results of the study itself, will be of great interest to clinicians and researchers.

Specific feedback/criticisms are follows:

1) Under "Psychotherapy" section of the Methods/Intervention it states that "For the purposes of this study, therapists are defined as staff with qualifications in a relevant health discipline (such as counseling, nursing, social work, psychology, psychiatry), including trainees with adequate skills that have undergone training on the use of the manual and in ketamine-assisted psychotherapy". More details are needed to describe what are the appropriate "qualifications" and "adequate skills". Also, it is questionable whether a "trainee" is an appropriate person to deliver this intervention: if authors are proposing to engage students as interventionists there needs to be explanation of the proper training and oversight provided to them. Also authors should provide a reference for the manual or consider including it as supplementary material.

2) The authors have thoroughly detailed the timing of ketamine administration and CBT sessions (Figure 2 is helpful). However, more details are needed on the setting and protections. It appears that CBT will occur 1-2 days after ketamine is given. Will both occur in the same clinic-based setting? Also, what protections are in place to ensure that that there is no therapist misconduct? There have been cases of therapist sexual misconduct in research on psychedelics that have recently emerged (see Kruger DJ 2024) highlighting the need for ethics/safeguards for such research (see Pilecki B Harm Reduction Journal 2021). Given the potential dissociative effects of ketamine, the author should consider similar protections against misconduct (such as having more than one therapist present).

3) Will the participants be assessed for acute intoxication prior to ketamine administration and CBT? How will it be determined whether they can proceed with visits?

4) The primary goals/outcomes are assessments of feasibility. These are clearly spelled out under the outcome session. However, the thresholds for meeting feasibility for these outcomes is not stated. Some discussion of how these key primary outcomes will be evaluated is merited.

5) Secondary outcomes include measures of "preliminary efficacy", including "self-reported change in the past 28-days of MA use from baseline to week 5 (post treatment), week 8 (primary endpoint), week 12 and week 24". Is this measured with TLFB? Is so, please state in paper. Also, it may not be accurate to say these are measures of efficacy given that there is no control. Related to that, did the authors consider study design that included a control (such as CBT only), and if so, why was that not pursued?

6) Related to the above, why did the authors choose to make week 8 their primary endpoint? Rationale for choosing this time point would be helpful.

7) I believe that the discussion would benefit from considering the future implementation of this intervention and its limitations for scalability. Administering ketamine as SC rather than IV does modestly expand potential for scalability, yet having an intervention that must be delivered by a nurse (and requires a 2-hour monitoring period after administration for safety) poses challenges for delivery in real-world settings both in terms of infrastructure/staffing and costs.

8) The paper uses a large number acronyms: recommend restricting use.

9) The sentence in the introduction 2nd paragraph stating “poorer outcomes observed in people who use methamphetamine frequently compared to those who use less frequently” needs citation.

Reviewer **2**

Name **Eaton, Erica**

Affiliation **Center for Alcohol and Addiction Studies, Brown University, Providence, RI, USA**

Date **13-Nov-2024**

COI **None**

Comments for the attention of the Authors:

While the authors have some minor modifications to make, the manuscript overall is very well written and is an excellent and needed addition to the existing literature. I recommend a few minor revisions and suggestions (see below) that I hope will serve to improve the paper:

Introduction:

• The introduction is very thorough, and well written.

• Minor edit: P. 9; Line 10 – I think the authors meant to say: “shown to sustain and improve treatment outcomes….”

• P. 9; Line 43 – please specify if the trials examining ketamine monotherapy for cocaine, heroin, and alcohol use utilized an active or standard/inactive placebo.

Participants and recruitment:

• P. 12; Lines 35-42: For length of time, it would be helpful to choice either number of weeks or days for consistency and ease of reading. In text, the timeline is in weeks and the figure is in days.

• P. 13; Lines 8-10: Unclear what the rationale is for the WURS…can the authors provide some context?

Intervention:

• P. 14, Can you provide specifics on the training the therapists received both for the use of the CBT manual and ketamine-assisted psychotherapy.

• P. 14, please provide more details regarding what happens on dosing day. Are the participants with their assigned therapist, under nursing supervision – who is in the room during ketamine administration? Is there any therapeutic support – if not, what is the rationale? Is there any attention paid to set and setting?

• P. 14 – please describe the 4-session CBT manual in more detail as a CBT approach includes a plethora of skills. It may be helpful to include topics/skills/content in each of the four sessions.

Thank you for the opportunity to review this critical and timely paper.

VERSION 1 - AUTHOR RESPONSE

Reviewer: 1
Dr. Judith Tsui, University of Washington, University of Washington School of Medicine

Comments to the Author:
This is a protocol paper describing an open label, single arm clinical trial of subcutaneous (SC) ketamine combined with cognitive behavioral therapy (CBT) weekly for one month for treatment of methamphetamine use disorder. Main outcomes are safety and feasibility measured over an 8-week period; secondary outcomes (including methamphetamine use, craving, withdrawal, quality of life and treatment satisfaction). The study addresses a clinical area of high need: methamphetamine use is a problem world-wide and effective treatments are needed. The focus on ketamine is timely. There are currently no regulatory-approved pharmacologic treatment options for methamphetamine use disorder (MUD) and there is increasing interest in ketamine as treatment for a wide variety of mental health and substance use disorders. The authors provide rationale to study ketamine for MUD given that it leads to dopamine depletion and depression and that there are a handful of small studies a ketamine monotherapy for depression, cocaine, heroin and alcohol use that suggest benefit. They provide rationale for combining with CBT arguing for ketamine-induced neuroplasticity that may enhance effects of CBT. The study design is well-conceived and clearly described, although there are some questions about the selection of the outcomes that need clarification. The protocol is detailed and there are helpful figures and tables to organize details of the study and orient the reader. One major limitation pertains to the delivery of the intervention and assurance of safety/comfort of research participants. Additionally, there are minor criticisms of writing and framing of the study intervention and its potential for scalable. Overall, criticisms should be addressable. I believe that

this study protocol, and eventually results of the study itself, will be of great interest to clinicians and researchers.

Specific feedback/criticisms are follows:
1) Under "Psychotherapy" section of the Methods/Intervention it states that "For the purposes of this study, therapists are defined as staff with qualifications in a relevant health discipline (such as counseling, nursing, social work, psychology, psychiatry), including trainees with adequate skills that have undergone training on the use of the manual and in ketamine-assisted psychotherapy". More details are needed to describe what are the appropriate "qualifications" and "adequate skills". Also, it is questionable whether a "trainee" is an appropriate person to deliver this intervention: if authors are proposing to engage students as interventionists there needs to be explanation of the proper training and oversight provided to them. Also authors should provide a reference for the manual or consider including it as supplementary material.

Response: We have updated the protocol and paper to remove reference to trainees; the therapist team are experienced clinicians who have delivered CBT in this population and have received additional training in ketamine-assisted components of the proposed approach. A therapist manual has been developed outlining this approach (unpublished and will be updated throughout the course of the pilot so therefore not included as supplementary material at this stage), and the paper has been revised to clarify this. The reference for the CBT manual is provided, and the ketamine-specific adaptations are outlined in the paper (see p12). A foreseen outcome of the pilot is an integrated manual combining CBT and ketamine-assisted components.

2) The authors have thoroughly detailed the timing of ketamine administration and CBT sessions (Figure 2 is helpful). However, more details are needed on the setting and protections. It appears that CBT will occur 1-2 days after ketamine is given. Will both occur in the same clinic-based setting? Also, what protections are in place to ensure that that there is no therapist misconduct? There have been cases of therapist sexual misconduct in research on psychedelics that have recently emerged (see Kruger DJ 2024) highlighting the need for ethics/safeguards for such research (see Pilecki B Harm Reduction Journal 2021). Given the potential dissociative effects of ketamine, the author should consider similar protections against misconduct (such as having more than one therapist present).
Response: CBT and ketamine administration are delivered in the same clinic-based setting, delivered by the therapist and/or the registered nurse (sometimes both present in the clinic room with the client). All clinic staff have trauma-informed care training and therapists are registered with professional bodies that adhere to strict ethical guidelines. Additionally, therapists are trained and supervised by a senior therapist with expertise in this area, including the 'set and setting' components of this approach with regards to establishing a safe context for clients (detailed in the therapist manual). We note that we are using low-dose ketamine which induces a transient response for up to two hours within a clinic-based setting where multiple practitioners are present, presenting minimal safety risks. This context differs from psychedelic-assisted therapies involving longer-acting substances (e.g., Psilocybin) and requiring extensive therapist involvement for up to 8 hours.

3) Will the participants be assessed for acute intoxication prior to ketamine administration and CBT? How will it be determined whether they can proceed with visits?
Response: A number of safety assessments are administered prior to the ketamine administration,

including vital sign measurement, review of adverse events, and the assessment of suicidality and mania using validated scales (CSSRS and YMRS respectively). These measures are reviewed by the Principal Investigator (a physician or psychiatrist specialised in substance use disorders) for any safety issues (such as the effects of acute intoxication) prior to administration. Therapists delivering the psychotherapy component are experienced clinicians working in a specialist methamphetamine clinic and will refer any safety concerns to the Principal Investigator.

4) The primary goals/outcomes are assessments of feasibility. These are clearly spelled out under the outcome session. However, the thresholds for meeting feasibility for these outcomes is not stated. Some discussion of how these key primary outcomes will be evaluated is merited.

Response:

We appreciate the reviewer's feedback and carefully considered it in our study design. As this is the first study of its kind in this population, we purposefully did not set specific feasibility thresholds due to the lack of comparable studies in this area (and therefore no benchmark to compare against) and the inherent biases of a single-site open-label pilot trial. Setting such thresholds prematurely could lead to incorrect assumptions about the study's success or failure in this context (Leon et al., 2011; Eldridge et al, 2016). For example, it will remain unknown how the study would perform in regional, remote or under-serviced areas, or with different therapists. Instead, we aim to present descriptive results (and qualitative acceptability; O'Cathain et al., 2015) to support decision-making for future larger studies. Nonetheless, we have taken on board the reviewer's feedback and added more clarity around each feasibility outcome to ensure transparency and facilitate progression to a definitive clinical trial. Specifically, the pilot study seeks to determine the (1) time taken to recruit the sample (with a 12-month study timeframe imposed, based on unpublished studies from our group successfully recruiting 20 participants within this timeframe); (2) proportion of ineligible participants at pre-screening and screening (eligibility criteria may need to be altered during the course of the pilot as required to ensure adequate patient flow is achieved within the 12-month study timeframe, however it is premature to provide metrics at this early stage as this is the first study implementing a stringent set of exclusion criteria for study entry in this population); (3) number receiving three doses of ketamine (no metric has been set as this is the first study to offer ketamine in conjunction with psychotherapy to those with methamphetamine use disorder. We will report descriptively all participants, and the number of doses received); (4) number completing 4 sessions of psychotherapy (we have not set a metric for success however we anticipate a 50% completion rate based on two studies conducted by Baker and colleagues (Baker et al., 2001, 2005), implementing 4-session CBT in this population. However, this was not ketamine-assisted psychotherapy and is therefore not directly comparable); (5) retention rate (we have not set a metric for success however we anticipate 75% completion of 6-month follow-up assessments as per Baker and colleagues (Baker et al., 2001, 2005), again noting that these studies are not directly comparable).

5) Secondary outcomes include measures of "preliminary efficacy", including "self-reported change in the past 28-days of MA use from baseline to week 5 (post treatment), week 8 (primary endpoint), week 12 and week 24". Is this measured with TLFB? Is so, please state in paper. Also, it may not be accurate to say these are measures of efficacy given that there is no control. Related to that, did the authors consider study design that included a control (such as CBT only), and if so, why was that not pursued?

Response: Self-reported MA use is measured via the TLFB, which has now been clarified in the paper (p14). We have taken care to frame these as measures of "preliminary efficacy and potential

mediators” and have amended the text as we recognise that efficacy can only be determined as part of a controlled study design. We opted not to include a control group in this pilot study as the key aim is to first establish safety and feasibility of this novel approach within this population. Study results will inform intervention refinement with a view to progressing toward a randomised controlled trial.

6) Related to the above, why did the authors choose to make week 8 their primary endpoint? Rationale for choosing this time point would be helpful.
Response: An 8-week primary endpoint was chosen as this allows one month post treatment for consolidation of psychotherapy (aligning with conventional timepoints in psychotherapy trials generally falling within 2-4 weeks post treatment). This additionally allows for comparison of self-reported MA use via TLFB from baseline (one month of data prior to commencing treatment) to follow-up (one month of data post-treatment) and adequate time to assess safety. The feasibility of this timepoint will be assessed as part of this pilot to inform a later randomised controlled trial.

7) I believe that the discussion would benefit from considering the future implementation of this intervention and its limitations for scalability. Administering ketamine as SC rather than IV does modestly expand potential for scalability, yet having an intervention that must be delivered by a nurse (and requires a 2-hour monitoring period after administration for safety) poses challenges for delivery in real-world settings both in terms of infrastructure/staffing and costs.

Response: We have expanded the discussion on scalability in the discussion, recognising the implementation challenges of medication-assisted psychotherapy interventions. Qualitative data from this study will be used to describe the acceptability and feasibility of the components which may allow for easier scalability (subcutaneous route, manualised psychotherapy). While out of the scope of this pilot study, we have noted the need for economic analyses in future studies.

8) The paper uses a large number acronyms: recommend restricting use.

Response: The paper has been updated accordingly to reduce the number of acronyms.

9) The sentence in the introduction 2nd paragraph stating “poorer outcomes observed in people who use methamphetamine frequently compared to those who use less frequently” needs citation.
Response: We have added a reference (Hillhouse et al., 2007) to support this statement.

Reviewer: 2
Dr. Erica Eaton, Center for Alcohol and Addiction Studies, Brown University, Providence, RI, USA

Comments to the Author:
While the authors have some minor modifications to make, the manuscript overall is very well written and is an excellent and needed addition to the existing literature. I recommend a few minor revisions and suggestions (see below) that I hope will serve to improve the paper:

Introduction:

(1) The introduction is very thorough, and well written.

(2) Minor edit: P. 9; Line 10 – I think the authors meant to say: "shown to sustain and improve treatment outcomes…."
Response: The paper has been updated accordingly.

(3) P. 9; Line 43 – please specify if the trials examining ketamine monotherapy for cocaine, heroin, and alcohol use utilized an active or standard/inactive placebo.
Response: The paper has been updated to clarify that these trials used an active placebo.

Participants and recruitment:
(4) P. 12; Lines 35-42: For length of time, it would be helpful to choice either number of weeks or days for consistency and ease of reading. In text, the timeline is in weeks and the figure is in days.
Response: Figure 1 has been updated in the paper to specify weeks rather than days.

(5) P. 13; Lines 8-10: Unclear what the rationale is for the WURS…can the authors provide some context?
Response: The WURS is often included in studies of methamphetamine use disorder given the disproportionate prevalence of childhood ADHD in this population (Bordoloi et al, 2019). While the sample size in this study is not sufficient to explore relationships between WURS scores and treatment outcomes (this will be examined in the later RCT subject to safety and feasibility being established in the current pilot), the WURS has been included to assist in characterization of the study sample and to allow comparisons to similar studies.

Intervention:
(6) P. 14, Can you provide specifics on the training the therapists received both for the use of the CBT manual and ketamine-assisted psychotherapy.
Response: The paper has been updated to clarify the training provided.

(7) P. 14, please provide more details regarding what happens on dosing day. Are the participants with their assigned therapist, under nursing supervision – who is in the room during ketamine administration? Is there any therapeutic support – if not, what is the rationale? Is there any attention paid to set and setting?
Response: The paper has been updated to clarify that a therapist manual has been developed specifically for this study outlining ketamine-assisted components of the intervention (and now briefly outlined in the paper). The therapist manual details what happens on dosing day (preparation discussion, intention setting, grounding exercises) and staff present during the first ketamine session (therapist will be present for the first 20-mins with the study nurse as part of intention setting and a therapeutic handover). Set and setting aspects are also detailed in the therapist manual.

(8) P. 14 – please describe the 4-session CBT manual in more detail as a CBT approach includes a plethora of skills. It may be helpful to include topics/skills/content in each of the four sessions
Response: A reference is provided in the paper for the 4-session CBT manual that outlines full details of the approach (Baker et al, 2003).

VERSION 2 - REVIEW

**Reviewer**	**1**
**Name**	**Tsui, Judith**
**Affiliation**	**University of Washington, Medicine**
**Date**	**06-Jan-2025**
**COI**	

The authors have provided detailed responses and made appropriate revisions to address the issues raised. I have no further comments/criticisms.

Thank you for the opportunity to review this manuscript and I look forward to study results.

**Reviewer**	**2**
**Name**	**Eaton, Erica**
**Affiliation**	**Center for Alcohol and Addiction Studies, Brown University, Providence, RI, USA**
**Date**	**08-Jan-2025**
**COI**	

Thank you for your attentive responses to the reviewers. I have no further comments.
